# Supplementary material for: Nitrite Determination in Environmental Water Samples Using Microchip Electrophoresis Coupled with Amperometric Detection
Source: Micromachines (Basel). 2022 Oct 14;13(10):1736. doi: 10.3390/mi13101736 (PMC9610075; doi:10.3390/mi13101736)
Supplement: Supplementary file 1 [file micromachines-13-01736-s001.zip › micromachines-1930133-supplementary.pdf]

SUPPORTING INFORMATION

Nitrite determination in environmental water samples using microchip electrophoresis coupled with amperometric detection

Simone B. Lucas[1], Lucas M. Duarte[1,2], Kariolanda C. A. Rezende[1] and Wendell K. T. Coltro[1,3]*

[1]*Instituto de Química, Universidade Federal de Goiás, Campus Samambaia, 74690-900, Goiânia, GO, Brazil.*

[2]*Instituto de Química, Departamento de Química Analítica, Universidade Federal Fluminense, Campus Valonguinho, 24020-141, Niterói, RJ, Brazil.*

[3]*Instituto Nacional de Ciência e Tecnologia de Bioanalítica (INCTBio), 13083-861, Campinas, SP, Brazil.*

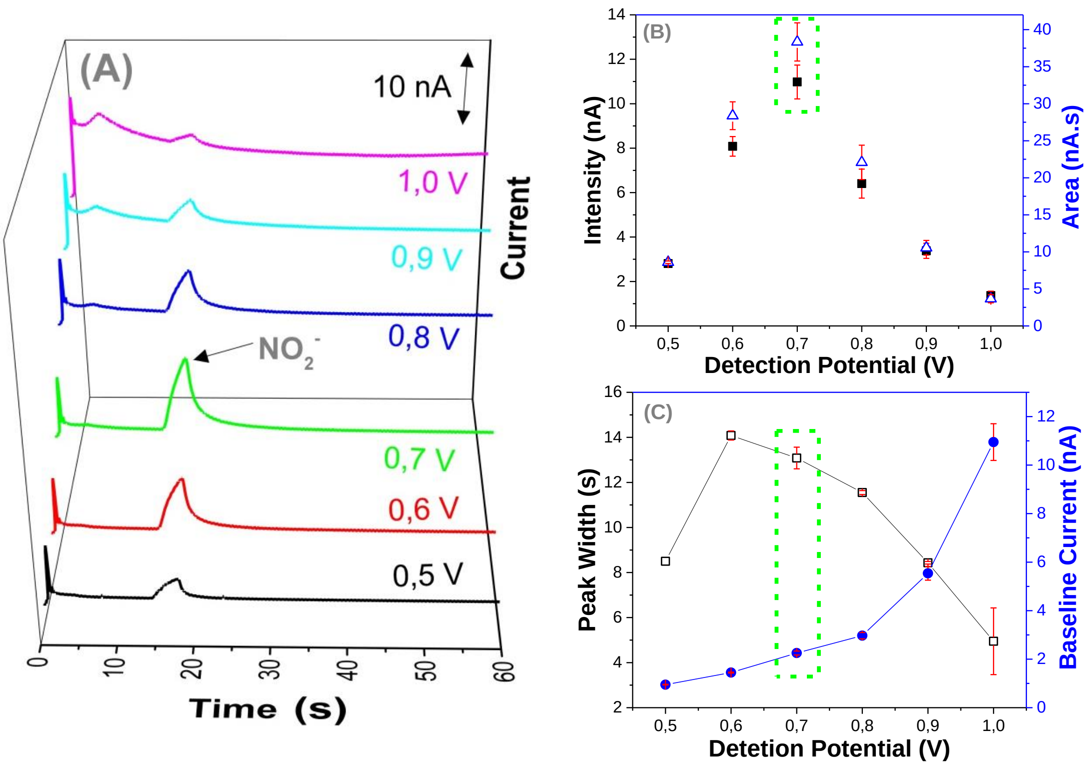

Figure S1. (A) Electropherograms showing the detection of nitrite (200 µmol L $^{-1}$) at different detection potentials (0.5 to 1.0 V *versus* Pt). **(B)** Peak Intensity and Area versus Detection Potential. **(C)** Peak width and Baseline current versus Detection Potential. Nitrite 200 µmol L^{-1}, Buffer: Lactic Acid/Histidine 30/15 mmol L^{-1}; Injection: -800 V for 10 s, Separation: -1000 V for 60 s.

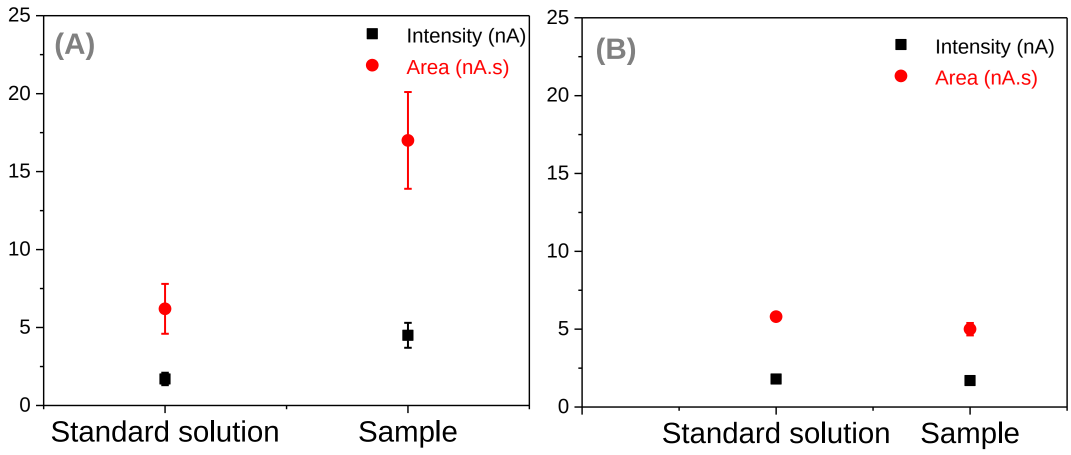

Figure S2. Comparison of nitrite detection with different dilution processes, in **(A)** dilution only in water, and **(B)** in 10% of background electrolyte. In both cases, the fortified sample and standard solution were prepared at the same concentration, 30 µmol L^{-1} of NO_2^-. Lactic Acid/Histidine Buffer 30/15 mmol L^{-1} pH = 3.8. Injection: -800 V for 10 s; Separation: -1000 V for 60 s, Detection: 0.7 V versus Platinum.
